# Detection and Feature Extraction in Acoustic Sensor Signals

**DOI:** 10.3390/s23198030

**Published:** 2023-09-22

**Authors:** Yuxing Li, Luca Fredianelli

**Affiliations:** 1 School of Automation and Information Engineering, Xi’an University of Technology, Xi’an 710048, China; 2 Shaanxi Key Laboratory of Complex System Control and Intelligent Information Processing, Xi’an University of Technology, Xi’an 710048, China; 3 Institute of Chemical and Physical Processes of National Research Council, Via G. Moruzzi 1, 56124 Pisa, Italy; luca.fredianelli@cnr.it

Our advances in detection and feature extraction in the processing of acoustic signals allow us to capture more information about a target and extract features with separability. Various trends suggest that detection and feature extraction play increasingly important roles in the processing of acoustic sensor signals. The eleven papers in this Special Issue and an Editorial signed by the book’s Editors cover a number of important topics and innovative approaches towards acoustic transducer signal processing, providing valuable techniques and ideas for related research.

The first work [1] in this volume focuses on seafloor Scholte waves. The authors proposed a method to detect these waves in an acoustic pressure field and found it to be effective even with sediment layers present. This study is important for understanding and detecting seafloor Scholte waves, and it provides valuable techniques for researching acoustic wave propagation in marine environments.

In reference [2], the authors proposed a deep learning hydroacoustic recognition method using the channel attention mechanism to address the issue of Doppler frequency shift in underwater targets caused by their motion speed and trajectory. The experimental results reveal that this method has significant advantages over other methods.

For conduit ranging and temperature measurements, the authors proposed an acoustic time-of-flight (TOF) estimation method based on digital lock-in filtering (DLF). The experimental results show the effectiveness of the proposed DLF method under different tube length and temperature conditions. The method has higher accuracy and robustness compared to conventional methods [3].

The fourth study proposes a delay estimation optimisation algorithm based on SVD and improved GCC-PHAT-ργ for efficient delay estimation under low signal-to-noise ratio conditions. The algorithm improves the signal quality via noise reduction and weighting methods and determines the delay difference using peak detection. The experiments confirmed that the algorithm significantly improved the delay estimation accuracy under a low signal-to-noise ratio with excellent performance [4].

Ascari, et al. [5] described methods to assess the effectiveness of low-noise pavements: the Close Proximity Index (CPX) methods allows measure noise near the wheels through sensors, while the Sound Pass-By (SPB) solves the problem through real-time events and roadside data feature extraction. The authors proposed a methodology improvement with the U-SPB, allowing to evaluate low-noise pavements in urban areas through unattended measurements and laboratory procedures. It considers long-term noise levels and traffic parameters.

Reference [6] describes the application of a partially updated adaptive algorithm PU to an extremely demanding structural active noise control ANC system to achieve global noise reduction and save computational power. The study discusses an improvement in the ANC system using the PU algorithm and verifies its high performance in the laboratory.

The seventh paper [7] proposes an FrFB-based inverse convolution beamforming method to improve the spatial resolution of DOA estimation by converting the signal to fractional-order Fourier domain via fractional-order Fourier transform, followed by delay and beamforming. The experimental results show that the method has higher resolution at low signal-to-noise ratios.

This study presents a new rolling bearing fault diagnosis method using dual-optimisation of WSO-HSlopEn and WSO-SVM [8]. By introducing HSlopEn as a new feature and dual-optimisation HSlopEn and SVM using WSO, the method demonstrates a high fault recognition rate in both single and multi-feature cases. The highest recognition rate is up to 100% [8].

For composite fault diagnosis of metro traction motor bearings, the authors used multi-signal fusion, MTF and optimised ResNet to improve both accuracy and effectiveness under complex working conditions. The method is able to extract the composite fault features under complex working conditions and improve the diagnostic accuracy and efficiency [9].

In the tenth [10] study presented in this paper, the authors proposed an adaptive parametric bearing fault detection method. By improving the grey wolf optimisation algorithm and optimising the structural parameters of the multi-stable stochastic resonance, the effective detection of bearing fault signals was achieved.

In order to improve the estimation accuracy problem of the cost reference particle filter (CRPF), the authors proposed an intelligent cost reference particle filtering algorithm based on multiple swarm co-operation. The simulation results show that the method has lower RMSE and MAE, reduces sensitivity to the initial values of the particles and improves the diversity of particles during resampling [11].
